# Burnout in International Medical Graduate Trainees in the United Kingdom Compared to Domestic Medical Graduate Trainees. Analysis of Data from the GMC’s National Training Survey in 2019 and 2021

**DOI:** 10.5334/pme.1036

**Published:** 2023-06-15

**Authors:** Mo Al-Haddad, Conal Mulholland, John Gardner

**Affiliations:** 1 Consultant in Critical Care and Anaesthesia at the Queen Elizabeth University Hospital, Glasgow, and Honorary Professor at the University of Glasgow, UK; 2 Clinical Teaching Fellow in Intensive Care Medicine at the Queen Elizabeth University Hospital, Glasgow, UK; 3 Consultant in Critical Care and Anaesthesia at the Queen Elizabeth University Hospital, Glasgow, UK

## Abstract

**Introduction:**

International Medical Graduates (IMGs) have lower educational attainment and a higher rate of complaints against them compared to Domestic Medical Graduates (DMG). The aim of this study was to investigate the potential role of burnout on these adverse outcomes experienced by IMGs.

**Methods:**

Every year, the General Medical Council (GMC) conducts the National Training Survey of all doctors in the United Kingdom which includes optional questions on work-related burnout from the Copenhagen Burnout Inventory (CBI). Work-related burnout data for doctors in training, linked to country of Primary Medical Qualification were obtained from the GMC for the years 2019 and 2021. Burnout scores of IMGs and DMGs were compared using Chi^2^.

**Results:**

The total number of eligible participants in 2019 and 2021 was 56,397 and 61,313 respectively. The response rates for all doctors in training to the CBI were 35,739 (63.4%) in 2019, and 28,310 (46.2%) in 2021. IMGs were at a lower risk of burnout compared to DMGs, 2,343 (42.9%) vs 15,497 (51.2%), Odds Ratio (OR) 0.72 (CI 0.68–0.76, P < 0.001) in 2019; and 2,774 (50.2%) vs 13,000 (57.1%), OR 0.76 (CI 0.71–0.80, P < 0.001) in 2021.

**Discussion:**

IMGs, as a group, appear to be at a lower risk of work-related burnout compared to DMGs. Burnout is unlikely to be contributing to lower educational attainment and higher rates of complaints experienced by IMGs compared to DMGs.

## Introduction

International Medical Graduates (IMGs) comprise a high proportion of the medical workforce reaching over 40% in the United Kingdom (UK) [1]. They undergo significant stress especially during the initial period of migration and working in the host country [2]. According to *structural theory*, burnout occurs when individuals fail to cope with chronic work stresses. This failure of coping mechanisms leads to professional failure, low professional fulfilment and depersonalisation [3]. Stress in doctors can lead to burnout [4] and has been associated with lower exam scores [5] and poor performance [6]. IMGs have lower pass rates in postgraduate exams [7,8] and a higher rate of complaints against them compared to Domestic Medical Graduates (DMGs) [9,10]. In addition, a recently published systematic review and meta-ethnography by Al-Haddad and colleagues highlighted that the mental health of IMGs was under-researched [2]. It would therefore be of interest to investigate if burnout rates were higher in IMGs compared to DMGs. Could burnout in IMGs be a contributing factor to the lower pass rates and higher complaint rates they experience?

A qualitative study of 96 trainees in the UK suggested that burnout was higher amongst IMG doctors in training (trainees) compared to DMG trainees, and that it might contribute to the attainment gap observed between these two groups [11]. However, West and colleagues conducted the largest survey to date comparing, amongst other issues, burnout in IMGs vs DMGs training in internal medicine in the United States [5]. They used two validated single-item questions to assess burnout rates in the *emotional exhaustion (EE)* and *depersonalisation (DP)* domains of the Maslach Burnout Inventory (MBI). A 77% response rate from 21,132 potential respondents revealed that the reported burnout in either of the two domains was less common in IMGs compared to DMGs. Weidner and colleagues reported a similar trend in a survey of American Board of Family Medicine diplomates using a single-item question related to the *EE* domain of the MBI, response rate 52% (n = 1,617) [12]. On the other hand, Aalto and colleagues surveyed 1,292 IMGs and a random sample of 7,000 Finnish DMGs from the database of the Finnish Medical Association and received a response rate of 45% and 54% respectively [13]. A single item in the survey related to burnout, revealed that IMGs were more likely to report a ‘high risk of burnout’ compared to DMGs.

As part of the quality assurance process of training and education in the UK, the General Medical Council (GMC) has conducted an annual National Training Survey (NTS) since 2013 of all doctors in training posts in the UK as well as their trainers [14]. The survey is based around five themes; the learning environment and culture, educational governance and leadership, supporting learners, supporting educators, and developing and implementing curricula and assessments [14]. In 2018, the GMC introduced in the NTS the seven items of *work-related burnout* from the Copenhagen Burnout Inventory (CBI) [15]. According to the CBI, work-related burnout is “*The degree of physical and psychological fatigue and exhaustion that is perceived by the person as related to his/her work*.” [15] The CBI has been validated in a wide range of settings including in health care professionals [15,16]. Using the work-related burnout scores from the NTS, Graham-Brown and colleagues demonstrated that IMG trainees in renal medicine had a higher risk of burnout compared to DMG trainees, but the trend was reversed for trainees in other medical specialties [17]. We searched both Pubmed and PsycINFO and did not identify a large national survey of burnout in IMG trainees in the UK nor one that compared burnout in IMG trainees to those of DMG trainees. Our research question was therefore: how do the reported burnout scores of IMG trainees in the UK compare to those of DMG trainees?

## Methods

All trainees in the UK are encouraged by the GMC to fill in the NTS annually. Due to the Covid-19 pandemic, the NTS was not carried out in the usual way in 2020. We were therefore interested in analysing data from the surveys of 2019 and 2021.

The methods of the surveys are published by the GMC in briefing notes [18,19]. In summary, a trainee is defined as being in one of the following posts: ‘*Foundation trainees, core trainees, higher specialty trainees including specialist registrars and GP trainees, Fixed term specialty training appointment trainees, locum appointment for training trainees, military trainees, trainees in clinical lecturer and academic clinical fellowship posts approved by the GMC, trainees working for non-NHS organisations (e.g., occupational medicine, pharmaceutical medicine and palliative medicine), trainees on out of programme trainees on an approved UK programmes at another deanery or Health Education England local office, post-certificate of completion of trainees training towards a sub specialty, Foundation 2 trainees completing additional GP training’*. Trainees were excluded if they were *‘on maternity or paternity leave on the census date, as well as trainees out of programme outside the UK, out of programme research, out of programme clinical experience or out of programme career break’* [18,19]. Before the survey, the GMC obtained data related to the trainees from the 18 Postgraduate Deaneries in the UK. From March 1–March 19, 2019, and from the April 1–April 19, 2021, the GMC conducted a pre-survey validation of data on trainees in 2019 and 2021 respectively. The surveys were distributed via email and were live between March 19–May 1, 2019, and from April 20-May 28, 2021. After completing the survey, each participant received a receipt, which training programs require as annual evidence of involvement in quality improvement.

Responding to the CBI’s seven questions of work-related burnout [15] is optional. The responses received are converted to a score from 0–100. The GMC defines burnout categories of low, medium and high if they were associated with score ranges of >50, 25–50, and <25 respectively [20].

In September 2021, the lead author requested NTS data from the GMC for the years 2019 and 2021 to answer the following questions: What was the response rate to the CBI section of the NTS for IMGs compared to DMGs? Was there a difference in reported burnout levels between IMGs and DMGs? If there was a difference, was this maintained throughout years of training? What were the reported burnout levels in IMGs and DMGs across the various specialties? What were the reported burnout levels in IMGs and DMGs across the four nations of the UK? If there was a difference in reported burnout levels, how did the pattern in 2019 compare to that of 2021?

The data received from the GMC were in absolute numbers and percentages according to the country of Primary Medical Qualification (PMQ), i.e. (IMG vs DMG). Responses were categorised as either low, moderate or high risk of burnout. When the number of responses for a certain category was less than three, no data were provided. For example, we did not receive burnout scores from training programmes with a small number of trainees, namely: broad based training, occupational medicine, and public health. We did not consider these training programmes for our analysis.

The GMC published the results of the NTS for 2019 and 2021, including the results of the burnout questionnaire [14]. However, our aim was to further analyse the data on burnout according to the trainees’ country of PMQ.

All data were categorical. We compared data using Chi^2^ and Odds Ratio (OR) (with 95% confidence intervals) tests using R [21]. As we were interested in whether there was a difference in reported risk of burnout, the categories of ‘medium’ and ‘high’ were considered as one category for the purpose of comparing risk of burnout between IMG trainees and DMG trainees. We therefore compared low vs (medium or high) risk of burnout in our analysis and considered a P value of < 0.05 to be statistically significant. However, to reduce the risk of type 1 error when running multiple statistical tests/sub-group comparisons, we applied the Bonferroni Calculation; adjusted P value= original P value/k (where k is the number of statistical tests run) [22]. Given the number of sub-groups (k) was equal to 27 (year of training, training programme, and home country) our adjusted P value = 0.05/27 = 0.002 (to three decimal places).

The NTS falls under the GMC’s responsibility of maintaining standards and requirements for postgraduate education and training [23]. Therefore, and in accordance with the UK’s Health Research Authority [24], the GMC data team confirmed that no ethical approval was required to carry out the survey. To guarantee anonymity, the GMC assures participants in advance that it would not share individual participant’s responses and would not publish data for a category or training programme if the number of responses was fewer than three [25].

## Results

We present the response rates to the NTS and the work-related burnout component of the CBI for both IMG trainees and DMG trainees in Table 1. We present burnout score categories of IMG trainees and DMG trainees in relation to the year of training, training programme, and home country in Tables 2, 3 and 4 respectively.

**Table 1:** Responses of International Medical Graduates (IMGs) and Domestic Medical Graduates (DMGs) to the National Training Survey (NTS) and *work-related burnout* component of the Copenhagen Burnout Inventory (CBI) in 2019 and 2021. Comparisons are between IMGs and DMGs.


	ALL TRAINEES N(%)	IMGS N(%)	DMGS N(%)	P VALUE OR (CI)

2019				

Total number of eligible trainees	56,397 (100)	9,780 (17.3)	46,617 (82.7)	

Response to NTS	53,477 (94.8)	9,052 (93.2)	44,425 (95.3)	<0.001* 0.61 (0.56–0.67)

Response to CBI	35,739 (63.4)	5,458 (55.8)	30,281 (65)	<0.001* 0.68 (0.65–0.71)

Risk of burnout				

Low	17,899 (50.1)	3,115 (57.1)	14,784 (48.8)	<0.001* 0.72 (0.68–0.76)

Moderate/High	17,840 (49.9)	2,343 (42.9)	15,497 (51.2)	

**2021**				

Total number of eligible trainees	61,313 (100)	13,351 (21.8)	47,962 (78.2)	

Response to NTS	46,795 (76.3)	9,815 (74)	36,980 (77.1)	<0.001* 0.82 (0.79–0.86)

Response to CBI	28,310 (46.2)	5,527 (41.4)	22,783 (47.5)	<0.001* 0.78 (0.75–0.81)

Risk of burnout				

Low	12,536 (44.3)	2,753 (49.8)	9,783 (42.9)	<0.001* 0.76 (0.71–0.80)

Moderate/High	15,774 (55.7)	2,774 (50.2)	13,000 (57.1)	


* Statistically significant at < 0.05.

**Table 2:** Reported levels of burnout in 2019 and 2021 for International Medical Graduates (IMGs) and Domestic Medical Graduates (DMGs) per year of training. Comparisons are between IMGs and DMGs.


	IMGS N (%)		DMGS N (%)		P VALUE OR (CI)
	
2019	LOW	MOD/HIGH	LOW	MOD/HIGH	

F1	85 (42.1)	117 (57.9)	2,137 (45.1)	2,597 (54.9)	0.39 1.13 (0.85–1.51)

F2	106 (41.7)	148 (58.3)	1,769 (40.8)	2,564 (59.2)	0.76 0.96 (0.75–1.25)

ST1/CT1	659 (56)	518 (44)	2,024 (47.6)	2,228 (52.4)	< 0.001* 0.71 (0.63–0.81)

ST2/CT2	477 (55.4)	384 (44.6)	2,027 (50.2)	2,008 (49.8)	0.006 0.81 (0.70–0.94)

ST3/CT3	737 (63.5)	423 (36.5)	2,288 (52.1)	2,102 (47.9)	< 0.001* 0.63 (0.55–0.94)

ST4	281 (56.7)	215 (43.3)	1,176 (49.7)	1,188 (50.7)	0.002 0.74 (0.61–0.90)

ST5	295 (55)	241 (45)	1,221 (53.3)	1,070 (46.7)	0.47 0.93 (0.77–1.13)

ST6	248 (60.3)	163 (39.7)	1,139 (54.6)	947 (45.4)	0.032 0.79 (0.64– 0.98)

ST7	175 (62.5)	105 (37.5)	776 (56.5)	597 (43.5)	0.065 0.78 (0.60–1.02)

ST8	52 (64.2)	29 (35.8)	227 (57.8)	166 (42.2)	0.28 0.76 (0.46–1.25)

Total	3,115 (57.1)	2,343 (32.5)	14,784 (48.8)	15,497 (51.2)	

**2021**					

F1	42 (39.6)	64 (60.4)	1,265 (38.2)	2,045 (61.8)	0.77 0.94 (0.63–1.40)

F2	81 (31.5)	176 (68.5)	1,100 (34.1)	2,125 (65.9)	0.4 1.12 (0.86–1.48)

ST1/CT1	654 (48.6)	693 (51.4)	1,317 (42)	1,815 (58)	< 0.001* 0.77 (0.68–0.87)

ST2/CT2	538 (51.1)	514 (48.9)	1,237 (41.4)	1,752 (58.6)	< 0.001* 0.67 (0.59–0.78)

ST3/CT3	593 (52.5)	537 (47.5)	1,453 (45.3)	1,758 (54.7)	< 0.001* 0.75 (0.65–0.86)

ST4	249 (48.9)	260 (51.1)	894 (46.9)	1,013 (53.1)	0.41 0.92 (0.76–1.12)

ST5	221 (49.2)	228 (50.8)	881 (46.3)	1,021 (53.7)	0.27 0.89 (0.72–1.09)

ST6	185 (52)	171 (48)	870 (52.5)	786 (47.5)	0.85 1.02 (0.81–1.29)

ST7	151 (57.4)	112 (42.6)	592 (52.5)	535 (47.5)	0.15 0.82 (0.63–1.08)

ST8	39 (67.2)	19 (32.8)	174 (53.7)	150 (46.3)	0.056 0.57 (0.31–1.02)

Total	2,753 (49.8)	2,774 (50.2)	9,783 (42.9)	13,000 (57.1)	


F1 and F2 = Foundation doctor at year 1 and 2 respectively, ST1-8 = Specialty Trainee (followed by year of training), and CT1-3 = Core Trainee (followed by year of training). * Statistically significant at p value < 0.002 using the Bonferroni correction.

**Table 3:** Reported levels of burnout in 2019 and 2021 for International Medical Graduates (IMGs) and Domestic Medical Graduates (DMGs) per training programme. Comparisons are between IMGs and DMGs.


	IMGS N (%)		DMGS N (%)		P VALUE OR (CI)
	
2019	LOW	MOD/HIGH	LOW	MOD/HIGH	

Foundation Programme	191 (41.9)	264 (58.1)	3,906 (43.1)	5,161 (56.9)	0.62 1.05 (0.87–1.27)

Acute Care Common Stem	40 (50)	40 (50)	406 (44.9)	498 (55.1)	0.38 0.82 (0.52–1.29)

Anaesthetics	132 (64.1)	74 (35.9)	1,324 (54.4)	1,110 (45.6)	0.007 0.67 (0.50–0.90)

Emergency Medicine	106 (45.3)	128 (54.7)	299 (35.7)	538 (64.3)	0.008 0.67 (0.50–0.90)

Medicine	605 (51.4)	573 (48.6)	2,098 (47.1)	2,353 (52.9)	0.01 0.84 (0.74–0.96)

Surgery	204 (60.4)	134 (39.6)	1,288 (51.9)	1,196 (48.1)	0.003 0.71 (0.56–0.89)

Obstetrics & Gynaecology	127 (53.6)	110 (46.4)	450 (42.3)	615 (57.3)	0.002 0.63 (0.48–0.84)

Paediatrics	234 (54.9)	192 (45.1)	914 (45.5)	1,095 (54.5)	< 0.001* 0.68 (0.56–0.85)

Ophthalmology	22 (57.9)	16 (42.1)	214 (64.5)	118 (35.5)	0.42 1.32 (0.67–2.61)

Radiology	83 (73.5)	30 (26.5)	553 (71.4)	221 (28.6)	0.66 0.90 (0.58–1.41)

Pathology	43 (76.8)	13 (23.3)	198 (81.5)	45 (18.5)	0.42 1.33 (0.66–2.68)

Psychiatry	313 (67)	154 (33)	744 (61.4)	468 (38.6)	0.03 0.78 (0.62–0.98)

General Practice	1,005 (62.1)	613 (37.9)	2,277 (52.6)	2,052 (47.4)	< 0.001* 0.68 (0.60–0.76)

**2021**					

Foundation Programme	123 (33.9)	240 (66.1)	2,365 (36.2)	4,170 (63.8)	0.37 1.11 (0.89–1.38)

Acute Care Common Stem	19 (32.2)	40 (67.8)	258 (36.4)	451 (63.6)	0.52 1.20 (0.68–2.12)

Anaesthetics	87 (53)	77 (47)	941 (48)	1,020 (52)	0.21 0.87 (0.59–1.12)

Emergency Medicine	88 (38.3)	142 (61.7)	248 (32.8)	508 (67.2)	0.13 0.79 (0.58–1.07)

Medicine	516 (45.4)	620 (54.6)	1,460 (43)	1,936 (57)	0.15 0.91 (0.79–1.04)

Surgery	144 (54.1)	122 (45.8)	803 (45.4)	967 (54.6)	0.008 0.70 (0.54–0.91)

Obstetrics & Gynaecology	121 (46.5)	139 (53.5)	324 (36.9)	553 (63.1)	0.005 0.67 (0.51–0.89)

Paediatrics	248 (53)	220 (47)	771 (51)	740 (49)	0.46 0.92 (0.75–1.14)

Ophthalmology	18 (50)	18 (50)	141 (52.4)	128 (47.6)	0.76 1.10 (0.55–2.21)

Radiology	62 (62)	38 (38)	414 (63.5)	238 (36.5)	0.77 1.07 (0.69–1.65)

Pathology	44 (68.8)	20 (31.2)	155 (72.1)	60 (27.9)	0.6 1.17 (0.64–2.15)

Psychiatry	263 (57)	198 (43)	566 (56.5)	435 (43.5)	0.86 0.98 (0.78–1.22)

General Practice	1,011 (53)	893 (47)	1,232 (41.4)	1,741 (58.6)	< 0.001* 0.63 (0.56–0.71)


* Statistically significant at p value < 0.002 using the Bonferroni correction.

**Table 4:** Reported levels of burnout in 2019 and 2021 for International Medical Graduate (IMGs) and Domestic Medical Graduates (DMGs) per home nation. Comparisons are between IMGs and DMGs.


	IMGS N (%)		DMGS N (%)		P VALUE OR (CI)
	
2019	LOW	MOD/HIGH	LOW	MOD/HIGH	

England	2,783 (57.1)	2,095 (42.9)	11,916 (48.2)	12,799 (51.8)	<0.001* 0.71 (0.66–0.75)

Northern Ireland	41 (47.1)	46 (52.9)	481 (48.1)	518 (51.9)	0.86 1.04 (0.67–1.62)

Scotland	168 (56.8)	128 (43.2)	1,758 (54.6)	1,461 (45.4)	0.48 0.92 (0.72–1.17)

Wales	123 (62.4)	74 (37.6)	629 (46.7)	719 (53.3)	<0.001* 0.53 (0.39–0.72)

**2021**					

England	2,417 (49.4)	2,456 (50.4)	8,076 (43)	10,707 (57)	<0.001* 0.77 (0.72–0.82)

Northern Ireland	66 (44.9)	75 (53.2)	311 (42.9)	414 (57.1)	0.39 0.85 (0.59–1.23)

Scotland	137 (55.5)	110 (44.5)	923 (43.3)	1,209 (56.7)	<0.001* 0.61 (0.47–0.80)

Wales	133 (50)	133 (50)	473 (41.4)	670 (58.6)	0.01 0.71 (0.54–0.92)


* Statistically significant at p value < 0.002 using the Bonferroni correction.

## Discussion

Our main findings were the lower levels of burnout in IMGs vs DMGs, and the lower response rate of IMGs compared to DMGs to the work-related CBI burnout questions. Our subgroup analyses revealed significantly lower burnout scores for IMGs vs DMGs at the Core Training (CT)/Specialty Training (ST)1 and CT/ST3 level of training, and in General Practice. In addition, there were consistently lower burnout scores for IMGs vs DMGs in England. However, in each of these subcategories, there was a larger number of respondents compared to the other subcategories. In addition, no subcategory demonstrated a reversal in the pattern of burnout between IMGs vs DMGs. Both observations suggest that the statistical significance achieved in the subcategory analyses was likely due to the larger number of respondents in these subcategories rather than a true difference in the burnout pattern.

The GMC has previously reported the higher burnout scores in 2021 compared to 2019 [14] which was likely to be due to the Covid-19 pandemic [26]. In our report, IMG trainees reported lower burnout scores compared to DMG trainees in both years.

The size of the sample and response rates, especially in 2019, make our report of the GMC’s work-related CBI dataset the largest comparing burnout in IMGs vs DMGs published yet. West and colleagues [5] used two single-item questions that were validated [27] against the EE and DP components of the MBI [28]. Weidner and colleagues [12] used only the question related to EE of the MBI that was used by West and colleagues [5]. Aalto and colleagues, on the other hand, used a single-item question on burnout that was not validated [13], this might have been part of the reason why their results were different to the previous two studies. Although the MBI is the most used measure of burnout, its continuous use has made it difficult to separate the measurement using MBI from burnout as a valid psychological construct [15,29]. In addition, both EE and DP are essential to the definition of burnout when using the MBI [30,31]. Even when West and colleagues reported burnout using two single-item questions [5], their definition of burnout was that respondents scored high on *either* EE or DP instead of defining burnout as scoring high on *both* EE and DP. This is a common issue in reporting and measuring burnout amongst doctors in training [29]. Our report, therefore, not only contains the largest number of participants, but also uses the largest number of validated items to measure burnout [15].

There is a plethora of evidence of a gap in the educational attainment between IMG trainees and DMG trainees [7,8,32]. Our findings indicate that the cause of this attainment gap -at group level- is unlikely to be due to burnout. This is not to say that burnout at an individual level is unassociated with poor performance during training and/or at postgraduate exams. Our results do suggest, however, that the focus of interventions to reduce burnout should be on identifying trainees who suffer or are at risk of suffering from burnout irrespective of their country of PMQ.

Further research in this area can be aimed at exploring the reasons behind a lower response rate to work-related burnout questions by IMGs compared to DMGs.

### Limitations

The first limitation of our study is the relatively low response rate (46.2%) to the CBI work-related burnout questions in 2021. Clearly, a low response rate puts the responses for 2021 at risk of reporting bias. Although this potential bias would have affected both groups, IMG trainees had a lower response rate compared to DMG trainees. This lower response rate might have led to an uneven under-reporting of burnout between the two groups. Since the percentage difference in response rates between IMG trainees and DMG trainees, and the percentage difference in reported levels of a low burnout score are very similar, it is theoretically possible that IMG trainees with a moderate or high level of burnout simply did not report it. However, the response rate to the CBI work-related burnout questions in 2019 was significantly higher (63.4%), but even then, the response for IMG trainees was lower than that of DMG trainees. It is unclear why IMGs were less likely to respond to the work-related burnout questionnaire and further investigation of this is warranted.

In addition to the two years of Foundation training in the UK which all medical school graduates undertake, specialty training takes between three to eight years to complete. There was, therefore, significant overlap between respondents in 2019 and those in 2021. Another limitation was that the data we obtained from the GMC were categorical. The GMC had already classified responses to burnout as either low, moderate, or high. Burnout is likely to be on a continuum rather than exist as a dichotomous entity [33]. Arguably, more continuous data would have improved the strength of our analysis. Finally, the CBI work-related burnout data were not linked to any other demographic factors, e.g., sex, age, or immigration status. Those links might have provided grounds for a more in-depth explanation of the results and provided an opportunity to explore associations between burnout and other demographic factors. However, the purpose of our enquiry was to investigate whether IMG trainees—as a group—reported different burnout levels to DMG trainees.

## Conclusion

IMGs, as a group, appear to be at a lower risk of work-related burnout compared to DMGs. Burnout in IMGs is unlikely to be contributing to the educational attainment gap and the higher rates of complaints experienced by IMGs compared to DMGs.
